# Foreign Body Ingestion in Children: A Narrative Review of Time-Critical Risk Stratification and Contemporary Management

**DOI:** 10.3390/children13081049

**Published:** 2026-08-06

**Authors:** Lăcrămioara Fodor, Gabriela Ghiga, Gabriela Păduraru, Nicoleta Gimiga, Laura Bozomitu, Bogdan Dragoș Rotaru, Elena Țarcă, Solange Tamara Roșu, Laura-Mihaela Trandafir

**Affiliations:** 1 Grigore T. Popa University of Medicine and Pharmacy, 700115 Iași, Romania; fodor.lacramioara@d.umfiasi.ro (L.F.); gabriela.ghiga@umfiasi.ro (G.G.); paduraru.gabriela@umfiasi.ro (G.P.); nicoleta.chiticariu@umfiasi.ro (N.G.); laura.bozomitu@umfiasi.ro (L.B.); solange.rosu@umfiasi.ro (S.T.R.); laura.trandafir@umfiasi.ro (L.-M.T.); 2 “Sf. Maria” Clinical Emergency Hospital for Children, 700309 Iași, Romania; bogdana_rtr@yahoo.com

**Keywords:** foreign body ingestion, children, time-critical management, risk stratification, button battery, multiple magnets, sharp objects, water beads, radiolucent foreign bodies, vulnerable pediatric patients

## Abstract

**Highlights:**

**What are the main findings?**

- Pediatric foreign body ingestion is usually benign, but button batteries, multiple magnets, sharp objects, and superabsorbent polymers require rapid risk stratification.
- Management should be adapted to the object type, location, symptoms, timing, and patient-specific risk factors.

**What are the implications of the main findings?**

- A structured, time-dependent approach may help prioritize endoscopic, surgical, or observational management in pediatric emergency settings.
- Radiolucent objects and vulnerable pediatric groups require a lower threshold for additional imaging, multidisciplinary assessment, and follow-up.

**Abstract:**

Background/Objectives: Foreign body ingestion is a frequent gastrointestinal emergency in children. Although most ingested objects pass spontaneously, button batteries, multiple high-powered magnets, sharp objects, and superabsorbent polymers may cause rapid and severe complications. Recent reviews have often focused on individual categories of high-risk objects, whereas practical integration of time-critical diagnosis, comparative guideline recommendations, radiolucent objects, vulnerable pediatric populations, and prognostic risk factors remains limited. This narrative review aims to critically synthesized current evidence and to propose a clinically oriented, risk-stratified framework that distinguishes emergent, urgent, and observational management pathways according to object characteristics, anatomical location, symptoms, elapsed time, imaging findings, and patient-related factors. Methods: A structured narrative review was performed using PubMed/MEDLINE, Scopus and Web of Science. The final searches were performed in July 2026 and covered publications indexed from January 2000 to June 2026. The review included international guidelines, position papers, systematic reviews, meta-analyses and clinically relevant observational studies in patients up to 18 years of age with gastrointestinal foreign body ingestion. Exclusion criteria were studies only involving adults, foreign body aspiration, nasal or auricular insertion, ingestion of caustic substances, duplicate publications, isolated case reports with no broader clinical relevance and articles not available in full text. Evidence was synthesized according to object type, anatomical location, urgency of intervention, imaging strategy, prognostic factors, and patient-related risk. Results: Most ingested foreign bodies pass spontaneously; however, button batteries, multiple magnets, sharp objects, and superabsorbent polymers are associated with distinct mechanisms of injury and a disproportionate risk of severe complications. Time to intervention, object type and size, anatomical location, symptom severity, delayed presentation, and imaging evidence of complications consistently emerge as the principal determinants of outcome. Current international recommendations show broad agreement for immediate removal of esophageal button batteries and sharp objects causing obstruction, but important differences remain regarding gastric batteries, distal magnets, radiolucent objects, and selected asymptomatic patients. Evidence for predictive models is still limited, and most proposed risk factors have not undergone external validation. Vulnerable children (e.g., with neurodevelopmental disorders, pica or recurrent intentional ingestion) require individualized multidisciplinary assessment and follow-up. Conclusions: Pediatric foreign body ingestion should be approached in a time-critical and risk-stratified manner, combining object characteristics, anatomical location, presenting symptoms, time from ingestion, imaging findings, and patient-specific vulnerability. Differentiated triage for emergent intervention, urgent removal and structured observation may improve clinical triage and reduce delays for high-risk cases. Current guidelines offer a robust management framework, but there are significant evidence gaps relating to radiolucent objects, water beads, distal magnets and externally validated predictive models. More multicenter studies are required to support standardized prognostic tools and more consistent decision-making across pediatric emergency settings.

## 1. Introduction

Foreign body ingestion is among the most frequent gastrointestinal emergencies in children, with the highest incidence occurring in those younger than five years. Although most ingested objects pass spontaneously and are associated with a favorable outcome, a clinically important subgroup requires urgent or emergent evaluation and intervention. This is particularly relevant in cases involving button batteries, multiple high-powered magnets, sharp or pointed objects, and complete esophageal obstruction, all of which are associated with an increased risk of severe complications [1,2,3].

The clinical significance of pediatric foreign body ingestion derives not only from the frequency of emergency department presentations but also from the potential for rapidly progressive and life-threatening complications. Esophageal necrosis, perforation, tracheoesophageal fistula, intestinal obstruction, and major vascular injury may occur, particularly after ingestion of button batteries, multiple magnets, or sharp objects. Recently, the increasing availability of small electronic devices, magnetic construction toys, and expandable recreational products has changed the epidemiological profile and risk spectrum of ingested foreign bodies, requiring continuous updates in diagnostic and therapeutic strategies [3,4,5].

The recent literature increasingly emphasizes the importance of rapid risk stratification based on object type, size, number, anatomical location, clinical presentation, and elapsed time since ingestion. These variables are essential for distinguishing situations that require immediate intervention from those that can be managed through urgent removal or structured observation. However, important areas of uncertainty persist, particularly in intermediate-risk scenarios, radiolucent foreign bodies, gastric button batteries, distal magnets, and asymptomatic patients, for whom international recommendations are not always fully concordant and the quality of evidence remains limited [1,2,3].

The aim of this narrative review is to critically synthesize current evidence on the diagnosis, risk stratification, and contemporary management of gastrointestinal foreign body ingestion in children. Particular emphasis is placed on time-critical clinical decision-making, comparison of international guideline recommendations, management of high-risk and radiolucent objects, prognostic factors, and the specific needs of vulnerable pediatric patients. The review also seeks to provide a practical framework for distinguishing emergent intervention, urgent removal, and structured observation in pediatric emergency care.

The novelty of this review lies in the integration, within a single clinically oriented framework, of time-critical risk stratification, explicit comparison of international society recommendations, management of radiolucent and emerging high-risk objects, evaluation of prognostic models, and the specific challenges posed by vulnerable pediatric populations. Unlike recent publications that have focused predominantly on individual categories such as button batteries, water beads, or specific complications [4,6,7,8], this review provides a broader synthesis across object type, anatomical location, elapsed time, imaging findings, and patient-related vulnerability. By organizing management into emergent, urgent, and observational pathways, the review aims to support more consistent and practical decision-making in pediatric emergency settings.

These high-risk objects produce distinct patterns of injury and diagnostic difficulty. Esophageal button batteries may cause severe tissue damage within a short interval; multiple high-powered magnets may lead to pressure necrosis, fistula formation, perforation, and intestinal obstruction, whereas superabsorbent polymers are frequently radiolucent and may produce delayed obstruction through progressive expansion. Therefore, confirmation of ingestion alone is insufficient; clinical decision-making must also integrate object type, number, and size; anatomical location; the child’s age and symptoms; and the time elapsed since ingestion [4,6,7,8].

Accordingly, this review proposes a clinically oriented conceptual framework based on the principles of time-critical management. The framework distinguishes between scenarios requiring emergent intervention, urgent removal, selected early intervention, structured clinical and radiological observation, or early surgical consultation. The accompanying flowchart organizes these principles into three sequential steps—initial assessment, risk stratification, and therapeutic decision-making. It was developed by integrating recommendations from major pediatric society documents and the reviewed literature and should be regarded as an illustrative decision-support framework rather than as a prospectively validated clinical algorithm.

## 2. Methodology (Literature Search Strategy)

This structured narrative review was informed by a structured and systematically conducted literature search focused on gastrointestinal foreign body ingestion in pediatric patients. The review was designed to provide a clinically oriented synthesis rather than a formal systematic review or meta-analysis.

The literature search was conducted in PubMed/MEDLINE, Scopus, and the Web of Science Core Collection. The final searches were performed in July 2026 and covered publications indexed between 1 January 2000 and 30 June 2026. The search strategy combined terms related to gastrointestinal foreign body ingestion, pediatric populations, high-risk object categories, diagnostic evaluation, risk stratification, and therapeutic management.

The following search concepts were combined using the Boolean operator AND:

(“foreign body ingestion” OR “ingested foreign body” OR “gastrointestinal foreign body” OR “esophageal foreign body”)

AND

(child OR pediatric OR paediatric* OR infant* OR adolescent*)**

AND

(“button battery” OR “button batteries” OR magnet OR “sharp object” OR “sharp objects” OR “superabsorbent polymer” OR “superabsorbent polymers” OR “water bead” OR “water beads” OR radiolucent OR endoscop OR surg* OR “risk stratification” OR management OR diagnos*).**

The search identified 634 records in PubMed/MEDLINE, 1293 records in Scopus, and 781 records in Web of Science, corresponding to a total of 2708 records. Search results were exported into a master screening database and deduplicated sequentially using the Digital Object Identifier, PubMed identifier, and normalized article title. Potential title-based matches that could not be confirmed automatically were retained for manual verification rather than being deleted. Fifteen Web of Science records could not be recovered from the supplied export and were documented as records removed for other reasons before screening. After removal of these records and 800 duplicate records, 1893 unique records remained for title-and-abstract screening.

Eligible publications included international clinical guidelines, position papers, consensus documents, systematic or scoping reviews, meta-analyses, pediatric cohort studies, diagnostic or prognostic studies, clinically relevant case series, and selected case reports when they illustrated rare but clinically important complications. Publications were eligible when they involved patients younger than 18 years or when pediatric data could be extracted separately.

Publications were excluded when they involved adults exclusively, foreign body aspiration without relevant gastrointestinal data, nasal or auricular foreign bodies, isolated caustic substance ingestion, publications outside the predefined date interval, duplicate publications, articles without clinically relevant diagnostic or management information, or full texts that could not be adequately assessed.

The 1893 unique records were evaluated according to their relevance to pediatric gastrointestinal foreign body ingestion, diagnosis, imaging, risk stratification, timing of intervention, endoscopic or surgical management, complications, and clinical outcomes. A total of 1862 records were excluded at the title-and-abstract screening stage because they did not directly contribute to the predefined clinical topics, represented lower-priority or overlapping evidence, or did not meet the eligibility criteria.

Thirty-one reports were sought for retrieval and assessed in full text. One adult-only ESGE guideline was excluded from the eligible pediatric evidence set but was retained as a contextual source for the explicit comparison of adult and pediatric recommendations. Therefore, 30 publications were included in the final narrative synthesis.

Evidence was synthesized narratively and organized according to object type, anatomical location, urgency of intervention, diagnostic imaging strategy, endoscopic or surgical management, prognostic variables, patient-related vulnerability, and areas of agreement or disagreement among international recommendations. No formal study-level risk-of-bias assessment, certainty-of-evidence grading, or quantitative meta-analysis was performed because of the narrative design and the substantial methodological heterogeneity of the available evidence.

**Figure 1:**
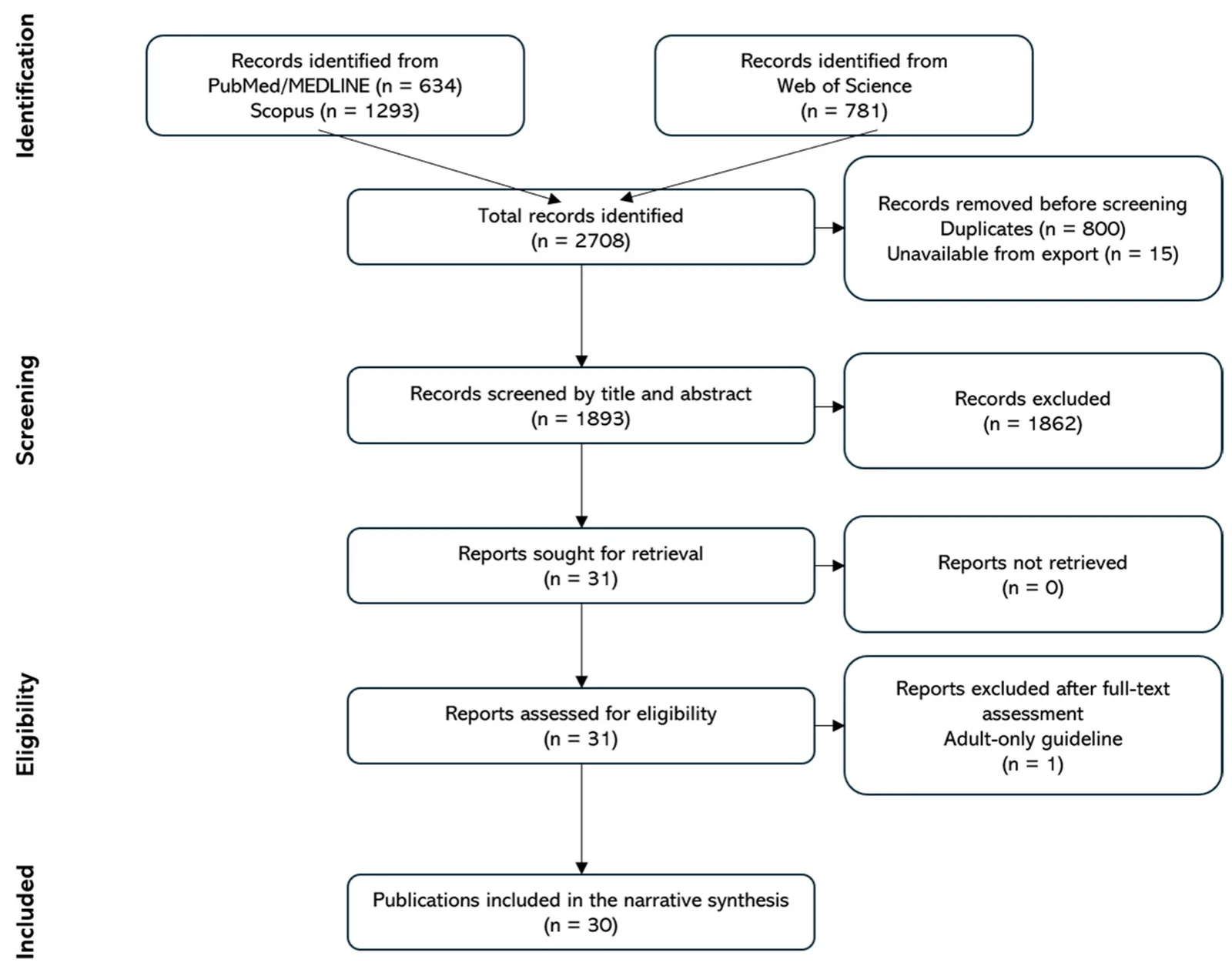
PRISMA-style flow diagram of literature identification, screening, eligibility assessment, and inclusion.

The literature-selection process is summarized in a PRISMA-style flow diagram (Figure 1). Because the manuscript was designed as a structured narrative review rather than a formal systematic review, the study-selection counts were retrospectively reconciled from the database exports, deduplication log, title-and-abstract relevance assessment, and final manuscript reference set. The PRISMA-style diagram was included to improve methodological transparency and should not be interpreted as evidence that the review followed all methodological requirements of a formal systematic review.

Because a formal certainty-of-evidence framework was not applied, the strength of the evidence was interpreted pragmatically according to study design, consistency across guidance documents, directness to pediatric practice, and the degree of agreement among major scientific societies. Recommendations were categorized narratively as reflecting broad guideline consensus, conditional guidance, observational evidence, expert consensus, or insufficient evidence. These categories were used to support transparent interpretation and should not be considered equivalent to formal evidence grading using GRADE or another validated framework.

## 3. Epidemiology and Clinical Impact

Foreign body ingestion is a frequent cause of presentation to pediatric emergency departments, particularly among children younger than five years. The highest incidence is reported between 6 months and 3 years of age, a developmental period characterized by active oral exploration, limited hazard awareness, and immature neuromotor coordination. Although both sexes are affected, several observational series have reported a slight male predominance [6,9,10].

Coins and other small blunt objects remain the most frequently ingested foreign bodies in children, and most pass spontaneously through the gastrointestinal tract without complications. Overall, approximately 80–90% of ingested foreign bodies pass without intervention, 10–20% require endoscopic removal, and fewer than 1% require surgical treatment. However, these proportions vary according to object type, anatomical location, patient age, clinical presentation, and the interval between ingestion and medical evaluation [1,6,11].

Over the past decade, the epidemiological profile of pediatric foreign body ingestion has evolved with the increasing availability of button batteries, high-powered magnets, and superabsorbent polymers such as water beads. Unlike conventional blunt objects, these products are associated with specific mechanisms of injury, delayed diagnosis, and a substantially higher risk of severe complications. Their ingestion may therefore require rapid endoscopic evaluation, close radiological surveillance, or early surgical involvement, depending on the object type, location, and clinical presentation [4,5,11].

The clinical impact of foreign body ingestion is highly variable and depends on the characteristics of the object, its anatomical location, the duration of impaction, and patient-specific factors. Although most children have a favorable outcome, high-risk objects may cause mucosal ulceration, esophageal necrosis, perforation, fistula formation, intestinal obstruction, gastrointestinal bleeding, and major vascular complications. Delayed recognition and prolonged tissue contact are particularly important determinants of morbidity [4,7,8].

From a healthcare system perspective, pediatric foreign body ingestion may require rapid access to diagnostic imaging; pediatric gastrointestinal endoscopy; surgical expertise; and, in complicated cases, intensive care support. Beyond the immediate clinical burden, hospitalization, repeated investigations, and prolonged follow-up may generate substantial resource use and psychological distress for children and their families. These consequences further support the need for early recognition, standardized risk stratification, and coordinated multidisciplinary management [6,7].

## 4. Diagnosis and Initial Assessment

The initial evaluation of a child with suspected gastrointestinal foreign body ingestion should be systematic, rapid, and directed toward three main objectives: confirming the ingestion when possible, determining the anatomical location of the object, and identifying early signs of complications. Clinical assessment should integrate history, physical examination, and imaging within a risk-stratified framework because management depends not only on the presence of a foreign body but also on its type, number, size, location, associated symptoms, and the time elapsed since ingestion [1,2,6]. The history should establish, whenever possible, the estimated time of ingestion, the suspected type of object, the number and dimensions of the ingested items, and whether the event was witnessed. Particular attention should be paid to symptoms such as dysphagia, odynophagia, drooling, chest or abdominal pain, vomiting, cough, respiratory difficulty, hematemesis, or refusal of food. In infants and young children, ingestion is frequently unwitnessed and may present with nonspecific symptoms; therefore, a high index of clinical suspicion is required, especially when the history is incomplete or inconsistent [1,6].

The physical examination should assess airway patency, respiratory status, ability to clear secretions, hydration status, and signs of gastrointestinal injury or obstruction. Warning signs include respiratory distress, stridor, persistent drooling, inability to swallow, severe chest or abdominal pain, hematemesis, gastrointestinal bleeding, abdominal distension, guarding, rebound tenderness, and other features suggestive of perforation or acute abdomen. If any of these signs are present, diagnostic and therapeutic management should be stepped up right away [1,2,6].

Conventional radiography remains the first-line imaging modality for suspected radiopaque foreign body ingestion. Anteroposterior and lateral views should be obtained to determine the number, size, orientation, and anatomical location of the object and to assess for indirect signs of obstruction or perforation. Radiographs are particularly important for differentiating a coin from a button battery: the “double-ring” or “halo” sign on the anteroposterior view and the “step-off” appearance on the lateral view are suggestive of a button battery and should prompt immediate evaluation for emergent removal [1,2,12].

Computed tomography should not be used routinely but may be indicated when complications are suspected or when the diagnosis remains uncertain after initial assessment. It is particularly useful in cases of suspected perforation, fistula formation, mediastinal or vascular injury, intestinal obstruction, or prolonged esophageal button battery impaction. The decision to perform CT should balance its diagnostic value against radiation exposure and should be individualized according to the child’s clinical condition and the suspected foreign body [2,4,12].

### Radiolucent Objects and the Limits of Radiography

Although anteroposterior and lateral radiographs are essential in the initial evaluation of suspected foreign body ingestion, their diagnostic performance depends largely on the material and location of the object. Radiopaque foreign bodies such as coins, button batteries, and most magnets are usually readily identified, whereas radiolucent objects may remain undetected despite a suggestive history or persistent symptoms. Consequently, a normal radiograph does not rule out foreign body ingestion and must always be analyzed in conjunction with clinical findings [1,2,12].

Common radiolucent foreign bodies include plastic and wooden objects, food boluses, thin fish or chicken bones, and superabsorbent polymers such as water beads. Because these objects may not be visible on conventional radiographs, diagnosis relies heavily on the clinical history, symptom pattern, and persistence of suspicion despite negative initial imaging. Particular caution is required in young children, in whom the ingestion may be unwitnessed and symptoms may be nonspecific [7,11,12].

When clinical suspicion persists despite normal or inconclusive radiographs, further imaging should be selected according to the suspected object and the child’s clinical status. Ultrasonography may identify some radiolucent objects and is particularly useful in young children because it avoids ionizing radiation. Computed tomography should be reserved for cases in which perforation, intestinal obstruction, fistula formation, or another complication is suspected, or when the diagnosis remains uncertain after initial clinical and radiographic assessment [1,2,7,12].

Water bead ingestion represents a particular diagnostic challenge because these superabsorbent polymers are generally radiolucent and may enlarge substantially after exposure to gastrointestinal fluids. As a result, the initial radiograph may be normal, while progressive expansion can later lead to partial or complete intestinal obstruction. Ultrasonography may help identify the beads or associated obstructive changes, whereas computed tomography is more useful when complications are suspected or the diagnosis remains uncertain. Because the evidence is based mainly on case reports and small case series, imaging and management should be individualized according to symptoms, object accessibility, and signs of progression or obstruction [11,13].

Accordingly, normal conventional radiographs should not be considered sufficient to exclude gastrointestinal foreign body ingestion when clinical suspicion remains high. Further evaluation should be individualized according to the suspected object, symptom persistence, anatomical location, and estimated risk of complications. In selected cases, additional imaging, diagnostic endoscopy, or surgical consultation may be required despite negative initial radiographic findings [1,2,7,12] (Table 1).

The differences between NASPGHAN and ESGE/ESPGHAN recommendations should not necessarily be interpreted as direct contradictions. The documents were developed at different times; used partially different evidence bases; and were intended for healthcare systems with variable access to pediatric endoscopy, anesthesia, advanced imaging, surgery, and specialized referral centers. In areas supported mainly by observational studies or expert consensus, these contextual differences may lead to different thresholds for additional imaging, endoscopic evaluation, or structured observation. Therefore, recommendations should be interpreted according to the clinical scenario, the strength and recency of the supporting evidence, and the resources available within the treating institution.

The practical contribution of radiography, ultrasonography, and computed tomography according to the suspected foreign body is summarized in Table 2.

The urgency of intervention and the degree of agreement among major international recommendations for high-risk ingestions are presented in Table 3.

Evidence interpretation: The recommendations summarized in this table are derived from society guidelines, position papers, retrospective observational studies, case series, and expert consensus. The degree of support varies across clinical scenarios. “Broad consensus” indicates substantial agreement among major guidance documents, whereas “conditional recommendation” reflects limited or heterogeneous evidence, dependence on patient or institutional context, or variation among society documents. These descriptors represent a narrative interpretation and are not formal GRADE certainty ratings.

Agreement is strongest for clearly time-critical situations, such as esophageal button batteries, sharp objects in the esophagus, complete obstruction, and symptomatic high-risk ingestions. Divergence is more frequent in intermediate-risk scenarios, including asymptomatic gastric button batteries, distal magnets, and selected objects beyond endoscopic reach. In these situations, differences among recommendations reflect limited comparative evidence; variable tolerance for procedural risk; and differences in the availability of repeated imaging, pediatric endoscopy, anesthesia, and surgical support.

## 5. Management According to Foreign Body Type

### 5.1. General Principles of Endoscopic Intervention

Proper preparation for endoscopic intervention is critical in the management of pediatric foreign body ingestion and directly impacts the success of the procedure and risk of complication. Preparation should be rapid but rigorous and should involve a multidisciplinary team including the pediatric gastroenterologist; anesthesiologist; and, in selected cases, the pediatric surgeon [1,2,6].

The first step is to establish the urgency of intervention—emergent, urgent, or non-urgent/observational—according to the type and location of the foreign body and the clinical status of the patient. Major endoscopic emergencies include esophageal button batteries, sharp objects in the esophagus, complete esophageal obstruction, and selected cases of multiple magnet ingestion or suspected perforation [1,2,4,5,8].

Before endoscopy, hemodynamic and respiratory stability, hydration status, aspiration risk, time elapsed since ingestion, and relevant comorbidities should be assessed. In unstable patients, stabilization takes priority over the endoscopic procedure. When perforation, complete obstruction, or major vascular injury is suspected, early multidisciplinary evaluation should be initiated without delaying definitive management [1,2,6].

In children, most endoscopic foreign body extractions are performed under general anesthesia with airway protection, particularly in high-risk situations such as esophageal button battery ingestion, bulky or sharp objects, marked drooling, complete obstruction, or a high risk of aspiration. Orotracheal intubation is generally preferred when secure airway control is required, as it reduces aspiration risk and facilitates endoscopic manipulation. Deep sedation may be considered only in carefully selected, clinically stable patients and in settings with appropriate pediatric anesthesia expertise [1,2].

### 5.2. Button Batteries and Cylindrical Batteries

Battery ingestion represents one of the most severe forms of gastrointestinal foreign body ingestion in children because of its potential to produce rapid tissue injury and life-threatening complications. Button batteries may cause mucosal damage through several mechanisms, including generation of an external electrolytic current, hydroxide formation at the negative pole, pressure necrosis, and leakage of battery contents. Deep esophageal injury may develop within a short interval after impaction, which justifies immediate intervention when a button battery is located in the esophagus [4,14,15].

The severity of injury is influenced by anatomical location, battery diameter and voltage, the child’s age, and the duration of mucosal contact. Lithium batteries measuring at least 20 mm are associated with a particularly high risk of esophageal impaction and severe injury in young children. An esophageal button battery is a time-critical emergency and should be removed immediately after diagnosis, ideally within 2 h. Removal should not be delayed because of fasting status or administration of mitigation therapy [1,4,14,15].

When ingestion has occurred within the previous 12 h, honey may be administered while the child is awaiting transfer or endoscopy, provided that the child is at least 12 months old, is clinically stable, and can swallow safely. The recommended dose is 10 mL every 10 min, for a maximum of six doses. Sucralfate may be administered in the medical setting, for a maximum of three doses. These measures are intended only to limit tissue injury and must never delay endoscopic removal. They are contraindicated when swallowing is unsafe, perforation is suspected, or the child is younger than 12 months in the case of honey [4,14,15].

After removal of an esophageal button battery, the mucosa should be carefully inspected and the depth and circumferential extent of injury documented. Because tissue damage may continue after extraction, subsequent management should be individualised according to the endoscopic findings and may include hospital admission, repeat endoscopic or imaging assessment, and multidisciplinary consultation. Delayed complications such as esophageal stricture, perforation, tracheoesophageal fistula, mediastinitis, or vascular fistula must be actively considered during follow-up [4,14,15].

Once a battery has passed beyond the esophagus, management becomes more individualized. Symptoms, battery type and diameter, the child’s age, time since ingestion, radiographic progression, and evidence of mucosal or intestinal injury should guide the need for observation, endoscopic removal, or surgical assessment. Recommendations for asymptomatic gastric button batteries are not fully uniform across NASPGHAN, ESPGHAN, and Poison Control guidance; therefore, no single removal threshold should be applied universally. Cylindrical batteries generally carry a lower risk of acute esophageal injury, but damaged batteries, persistent gastric retention, absent progression, or the development of symptoms require specialist reassessment. The main clinical scenarios and recommended management pathways are summarized in Table 4 [1,4,14,15].

### 5.3. Magnets

Ingestion of multiple high-powered magnets, or of a magnet together with another metallic object, represents a high-risk situation because attraction across adjacent bowel loops may produce pressure necrosis, fistula formation, perforation, obstruction, volvulus, or peritonitis. The severity of injury depends on the number of magnets, their anatomical location, the interval since ingestion, and whether they remain separated within different intestinal segments [1,5,7].

Anteroposterior and lateral radiographs are essential to determine the number, location, and apparent configuration of the magnets. However, several magnets adherent to one another may mimic a single object on radiography; therefore, apparent single-magnet ingestion should be interpreted cautiously when the history is uncertain. Serial imaging may be required to assess progression, but radiographic movement does not completely exclude ongoing attraction between bowel loops [1,5,7].

Management should be individualized according to magnet number, anatomical location, symptoms, radiographic configuration, and progression on serial imaging. Endoscopically accessible magnets generally require early removal, whereas multiple magnets beyond endoscopic reach warrant close surgical involvement because complications may develop despite an initially reassuring presentation. Observation is appropriate only in carefully selected asymptomatic patients with reliable follow-up and immediate access to reassessment. The main clinical scenarios and recommended management pathways are summarized in Table 5 [1,5,7].

### 5.4. Sharp Objects

Ingestion of sharp or pointed objects is associated with a substantial risk of gastrointestinal injury, including mucosal laceration, perforation, extraluminal migration, abscess formation, and injury to adjacent structures. Commonly ingested objects in children include needles, pins, toothpicks, fish or chicken bones, and metallic fragments with sharp edges. Management depends primarily on anatomical location, object dimensions and orientation, symptoms, elapsed time, and evidence of complications [1,2,6,11].

Sharp or pointed objects located in the esophagus require emergent endoscopic removal because of the risk of perforation and injury to adjacent mediastinal structures. Airway protection should be considered according to the child’s age, clinical condition, secretion handling, aspiration risk, and object characteristics. When extraction is performed, the sharp end should be oriented distally whenever technically feasible, and protective measures such as a retrieval net, protective cap, or overtube may reduce mucosal trauma. The mucosa should be carefully inspected after removal to identify laceration, ulceration, perforation, or bleeding [1,2].

Sharp objects located in the stomach or proximal duodenum generally require urgent endoscopic removal when they are accessible and extraction can be performed safely. The choice of retrieval device should be adapted to the shape and size of the object and may include rat-tooth or alligator forceps, snares, retrieval nets, or baskets. If safe endoscopic extraction is not possible, multidisciplinary discussion with pediatric surgery is required [1,2].

Once a sharp object has passed beyond the ligament of Treitz, management should be individualized. In an asymptomatic child without signs of complications, close clinical and radiographic surveillance may be considered. Surgical assessment is required if abdominal pain, vomiting, fever, gastrointestinal bleeding, obstruction, peritoneal signs, or radiographic lack of progression develops. Because the optimal duration and interval of monitoring are not uniformly established, these recommendations should be interpreted as conditional and based mainly on observational evidence and expert consensus [1,2,14]. Radiolucent sharp objects, including toothpicks, wooden fragments, and some thin fish bones, represent a particular diagnostic challenge. A normal radiograph does not exclude ingestion, and persistent symptoms or strong clinical suspicion may justify further imaging, diagnostic endoscopy, or surgical consultation. Diagnostic delay may increase the risk of perforation, migration, and localized or systemic complications [1,2,6,11].

### 5.5. Coins and Blunt Objects

Coins and small blunt objects are among the most commonly ingested foreign bodies in children. In most cases, the clinical course is favorable, and once a small, smooth, and inert object has passed beyond the esophagus, spontaneous passage through the gastrointestinal tract is expected without complications [1,2,7].

Management is determined primarily by anatomical location, object size, and the presence of symptoms. Esophageal coins in symptomatic children, particularly those with drooling, dysphagia, respiratory symptoms, or inability to handle secretions, require emergent endoscopic removal. In asymptomatic children, removal should be performed within 24 h if the coin remains in the esophagus; repeat radiography before endoscopy may be useful to confirm persistence or spontaneous passage [1,2,6].

Gastric coins and other small blunt objects in asymptomatic children can usually be managed by clinical observation and, when indicated, radiographic follow-up. Elective endoscopic removal may be considered if symptoms develop, if the object remains in the stomach for a prolonged period, or if its dimensions are unlikely to permit passage through the pylorus or duodenal sweep. Objects with a large diameter or excessive length require individualized assessment because age and body size influence the likelihood of spontaneous passage [1,2,6,14].

Radiographic differentiation between a coin and a button battery is essential because the urgency and management are fundamentally different. A double-ring or halo appearance on the anteroposterior view, together with a step-off appearance on the lateral view, should raise suspicion of a button battery and prompt immediate reassessment as a time-critical emergency [1,2,4,12].

### 5.6. Superabsorbent Polymers (Water Beads): An Emerging Diagnostic and Therapeutic Challenge

Superabsorbent polymers, commonly known as water beads, represent an emerging category of gastrointestinal foreign bodies whose clinical relevance has increased with their widespread use in sensory toys, educational materials, decorative products, and household items. Although ingestion is less frequent than that of coins or button batteries, the consequences may be severe, particularly in infants and young children [11,13,16,17].

The defining characteristic of these objects is their ability to absorb fluid and progressively expand to many times their initial size. After passing through the stomach, water beads may continue to enlarge within the small intestine, where they can cause partial or complete intestinal obstruction through luminal occlusion, distension, and local compression. In severe cases, bowel ischemia, perforation, and the need for surgical resection have been reported [11,12,13,16,17].

Diagnosis is challenging because water beads are generally radiolucent and are usually not visible on conventional abdominal radiographs. Therefore, a normal radiograph does not exclude ingestion. Careful history-taking and a high index of suspicion are essential, especially in young children with unwitnessed ingestion and progressive vomiting, abdominal pain, distension, or reduced intestinal transit. Ultrasonography may identify rounded, well-defined, hypoechoic or anechoic structures and associated signs of obstruction, whereas computed tomography should be reserved for selected cases in which the diagnosis remains uncertain or complications are suspected [11,12,13,16,17].

Management remains individualized because no universally accepted protocol or validated prognostic criteria are currently available. Clinical decisions should integrate age, symptoms, timing, estimated bead number and expansion capacity, suspected location, and evidence of progression or obstruction. Early gastroenterological assessment may be appropriate when recently ingested beads remain accessible in the upper gastrointestinal tract, whereas persistent symptoms, obstructive findings, or clinical deterioration require urgent surgical evaluation. The principal symptom- and location-based management scenarios are summarized in Table 6 [11,12,13,16,17].

**Figure 2:**
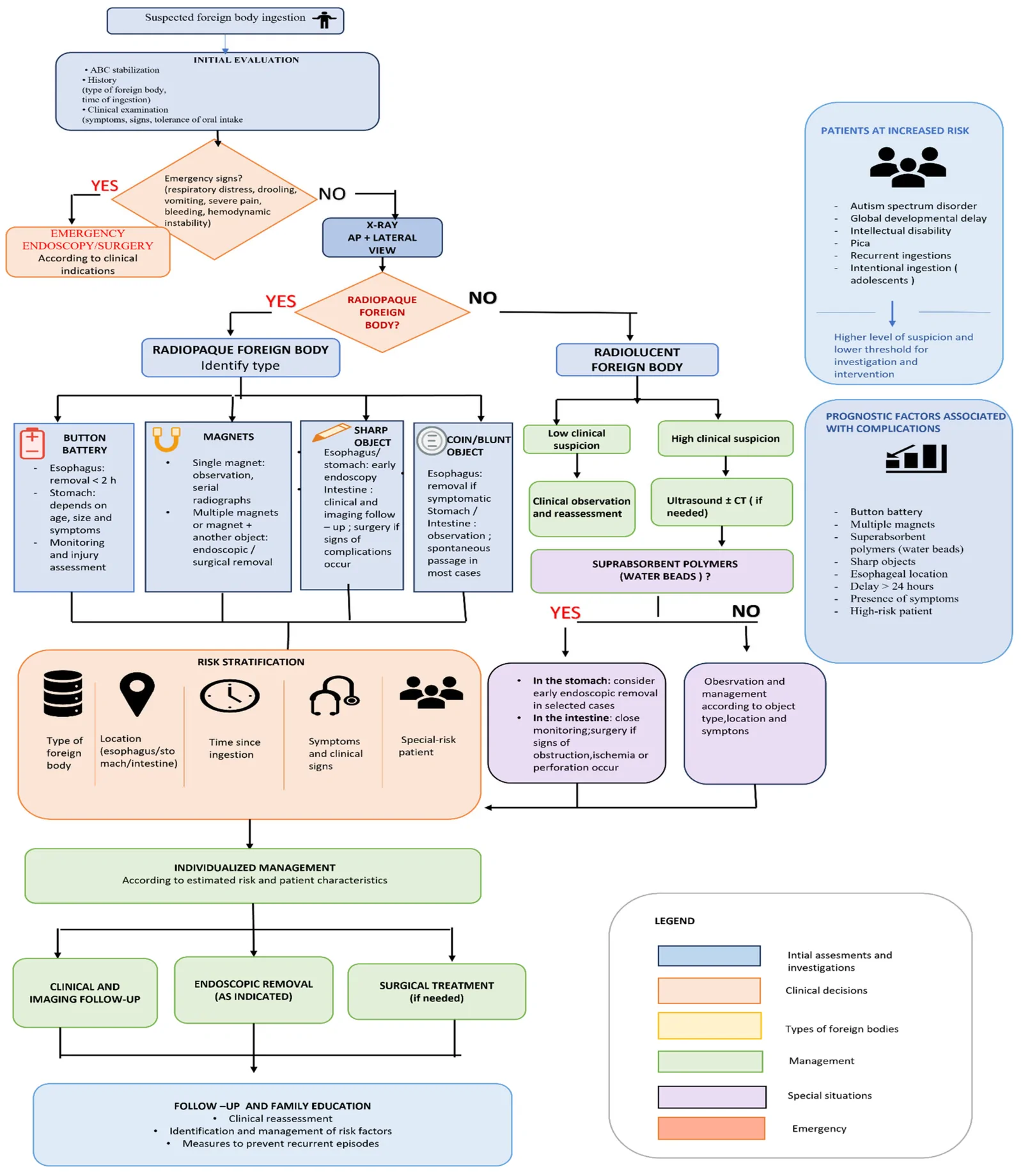
Proposed conceptual flowchart for the time-critical and risk-stratified management of gastrointestinal foreign body ingestion in children. The framework integrates initial clinical assessment, object characteristics, anatomical location, symptoms, imaging findings, elapsed time since ingestion, and patient-related vulnerability to distinguish emergent intervention, urgent management, selected early intervention, and structured observation. It was developed by synthesizing recommendations from NASPGHAN, ESGE/ESPGHAN, and specific ESPGHAN position papers and has not undergone prospective clinical validation [1,2,4,5].

Integrating the diagnostic and therapeutic principles discussed above, Figure 2 presents a proposed conceptual flowchart for pediatric gastrointestinal foreign body ingestion, progressing from initial assessment and risk stratification to time-critical and individualized management.

## 6. Prognostic Factors and Predictive Variables

The clinical course of gastrointestinal foreign body ingestion in children is determined by the interaction between object-related characteristics, anatomical location, patient-specific vulnerability, clinical manifestations, imaging findings, and the time elapsed before evaluation and intervention. Early recognition of high-risk features may help prioritize endoscopic or surgical assessment and support individualized management. However, most reported variables originate from retrospective observational studies and should not automatically be interpreted as independently validated prognostic factors [3,6,9,10,18,19,20,21].

Object-related characteristics are among the most consistently associated determinants of risk. Button batteries, multiple high-powered magnets or a magnet ingested together with another metallic object, sharp or pointed objects, and superabsorbent polymers are associated with specific mechanisms of tissue injury and a disproportionate risk of complications. Object size, shape, number, chemical composition, and expansion capacity may further influence the likelihood of impaction, mucosal injury, failure of progression, obstruction, or the need for invasive intervention [3,4,6,7,8,10,12,19,21].

Anatomical location substantially modifies the clinical risk. Esophageal impaction, particularly of a button battery or sharp object, is associated with rapid tissue injury and requires time-critical intervention. In the gastrointestinal tract, multiple magnets located across adjacent bowel loops may produce compression, ischemia, pressure necrosis, perforation, or entero-enteric fistula formation. Location should therefore be interpreted together with object type, radiographic configuration, progression on serial imaging, symptoms, and elapsed time [1,4,5,6,7,19].

Patient-related characteristics may also contribute to a complicated course. Young age, pre-existing disease, previous gastrointestinal surgery, anatomical or functional abnormalities of the gastrointestinal tract, and neurodevelopmental or behavioral disorders have been associated with delayed recognition, increased need for intervention, or complications in observational cohorts. In a pediatric study involving 772 patients, age younger than 3 years, pre-existing disease, foreign body type and location, and retention exceeding 24 h were reported as independent variables associated with complications in multivariable logistic regression [10,18]. These findings remain cohort-dependent and require validation in other populations.

Clinical manifestations at presentation are important for both risk assessment and therapeutic prioritization. Drooling, dysphagia, odynophagia, vomiting, retrosternal or abdominal pain, hematemesis, refusal of food, respiratory symptoms, and unexplained crying may indicate impaction, obstruction, or tissue injury. Several predictive models have incorporated symptoms together with patient characteristics and foreign body features [21,22].

Time is a major determinant of severity, although its prognostic meaning differs according to object type. For esophageal button batteries, longer mucosal contact is directly associated with deeper injury. For multiple magnets, delayed recognition and lack of progression may allow sustained compression between bowel loops and the development of ischemia, necrosis, fistula, or perforation. In broader pediatric foreign body cohorts, prolonged retention, including intervals exceeding 24 h in some studies, has been independently associated with complications. Nevertheless, a single time threshold should not be applied uniformly to all foreign bodies [4,5,10,18,21,23].

Imaging findings may strengthen prognostic assessment. Lack of radiographic progression, multiple magnets separated across different bowel segments, signs of obstruction or perforation, free air, bowel-wall abnormalities, or suspected involvement of adjacent structures indicates increased risk and should prompt escalation of care. Recent models focused on multiple magnet ingestion have incorporated clinical and radiological variables into nomograms intended to estimate the risk of complications. These models illustrate the potential value of combined clinico-radiological assessment but remain insufficiently externally validated for routine use [5,12,24].

Laboratory abnormalities should be interpreted as indicators of possible complications rather than as validated prognostic biomarkers of foreign body ingestion. Leukocytosis, elevated C-reactive protein, metabolic abnormalities, anemia, or other inflammatory changes may support suspicion of perforation, mediastinitis, peritonitis, intestinal ischemia, or sepsis in the appropriate clinical context. Normal laboratory findings do not exclude significant local injury, particularly during the early phase of presentation.

Recent research has shifted from isolated prognostic variables toward multivariable prediction models and clinical scoring systems. The multicenter nomogram reported by Mantegazza et al. was developed from 5864 pediatric cases and combined clinical symptoms, pre-existing disease, and foreign body characteristics to estimate the probability of endoscopic or surgical intervention. The model showed moderate discrimination, with an area under the receiver operating characteristic curve of approximately 0.77. However, the reported performance primarily reflects discrimination within the development population, and information regarding calibration, clinical utility, and independent external validation remains limited [21,22].

Other pediatric scoring systems have combined variables such as age, general clinical condition, symptoms, object type, size, and anatomical location. Although prospective evaluation in a single pediatric center suggests that structured risk assessment is feasible, these tools were derived or tested in restricted populations and have not been directly compared across independent cohorts or healthcare systems [24,25]. Differences in outcome definitions are also important: some models predict complications, whereas others predict the need for endoscopic or surgical intervention. These outcomes should not be considered interchangeable.

Consequently, existing models should be interpreted as exploratory decision-support instruments. Before routine clinical implementation, they require transparent reporting of model development, assessment of discrimination and calibration, evaluation of clinical utility, independent external validation, and comparison with guideline-based clinical judgment.

Accordingly, prognostic assessment should not rely on a single variable or on an unvalidated prediction score. A clinically meaningful estimate of risk requires integration of object characteristics, anatomical location, symptoms, patient-related vulnerability, imaging findings, laboratory indicators of complications, and elapsed time. Future model development should use standardized outcome definitions, appropriate internal validation, assessment of both discrimination and calibration, and independent external validation in multicenter pediatric populations before implementation in routine practice [22,24,25].

### Pediatric Patients at Increased Risk

Although most foreign body ingestions are accidental events occurring in previously healthy young children, several pediatric groups have an increased risk of unwitnessed ingestion, delayed diagnosis, recurrent episodes, intentional ingestion, and complications. Identification of these vulnerable populations should influence the threshold for investigation, the intensity of observation, multidisciplinary involvement, and planning for recurrence prevention.

Neurodevelopmental disorders, communication impairment, pica, psychiatric disease, intentional or recurrent ingestion, and pre-existing anatomical or functional gastrointestinal abnormalities may increase the likelihood of unwitnessed events, delayed recognition, repeated ingestion, impaction, and complications. These factors do not independently determine the need for intervention, but they may justify a lower threshold for imaging, specialist assessment, multidisciplinary involvement, mental health evaluation, and structured follow-up. The principal vulnerable pediatric groups and their specific clinical implications are summarized in Table 7 [26,27].

**Figure 3:**
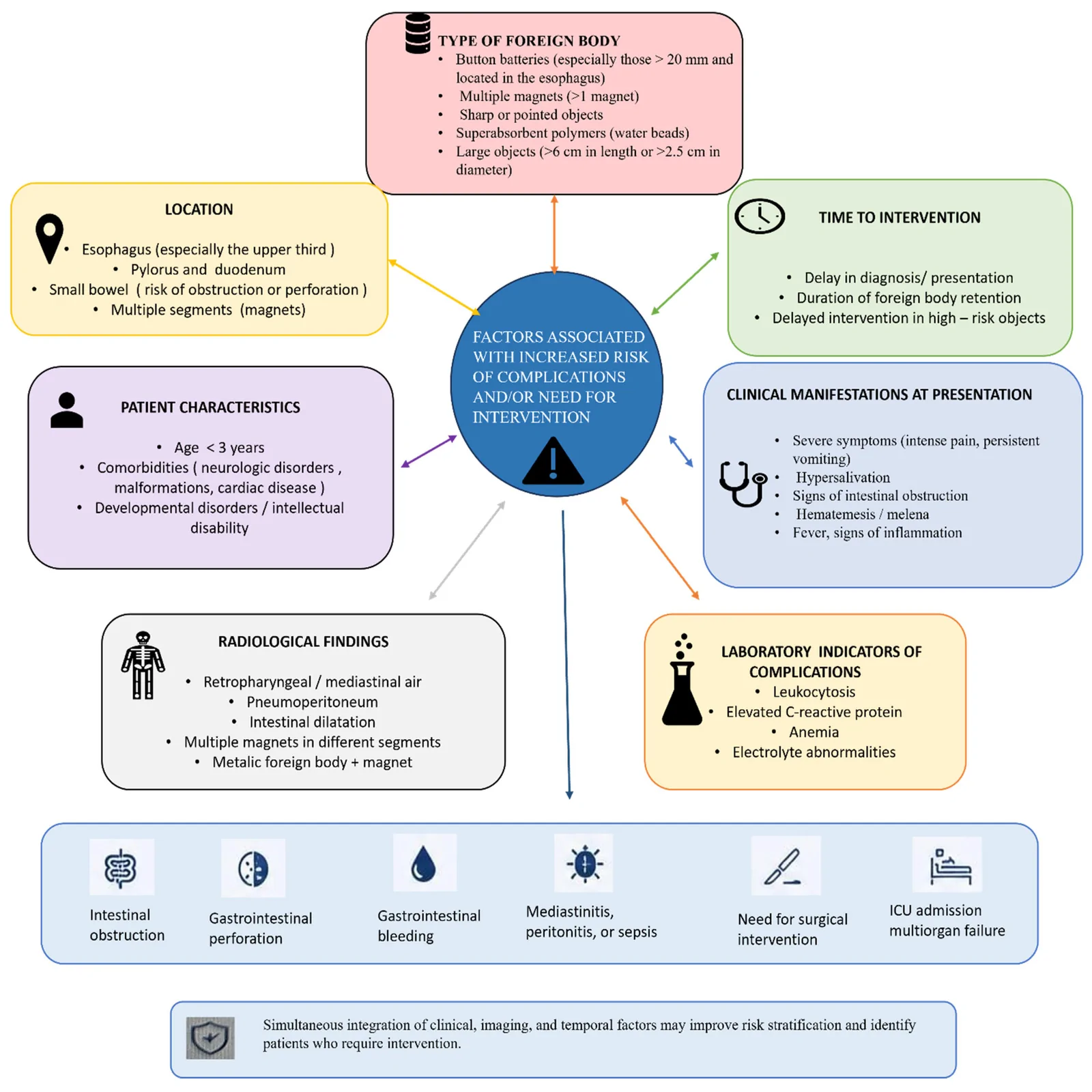
Main prognostic factors associated with complications in pediatric foreign body ingestion and their role in risk stratification and choice of therapeutic conduct [18,19,20,21,22,23,24,25].

The prognostic variables discussed in this section are integrated in Figure 3, which illustrates how object characteristics, location, time, clinical presentation, imaging findings, and patient-related factors contribute to complication risk and therapeutic decision-making.

## 7. Controversies, Evidence Gaps, and Current Research Directions

Despite substantial progress in the diagnosis and management of pediatric foreign body ingestion, clinically important areas of uncertainty remain. These controversies arise from differences between international guidance documents, the predominance of retrospective studies and case series, heterogeneous definitions of complications, variation in local access to pediatric endoscopy and surgery, and the practical difficulty of conducting randomized trials in emergency pediatric settings. Accordingly, recommendations should be interpreted according to their source, methodological basis, consistency across guidance documents, and applicability to the individual clinical and institutional context. In this review, statements are differentiated narratively as broad guideline consensus, conditional recommendations, observational associations, expert opinion, or areas of insufficient evidence; these terms should not be interpreted as formal certainty-of-evidence grades [1,2,4,5,7].

Differences among international recommendations may also reflect the methodological and organizational context in which each document was developed. NASPGHAN guidance was published earlier and provides a broad pediatric management framework, whereas later ESGE/ESPGHAN guidelines and object-specific ESPGHAN position papers incorporate more recent evidence and focus in greater detail on selected high-risk ingestions. Nevertheless, many recommendations remain based on retrospective cohorts, case series, and expert consensus rather than on direct comparative trials.

Resource availability is another important determinant of how recommendations are applied. A strategy requiring repeated radiography, urgent pediatric endoscopy, protected airway management, advanced cross-sectional imaging, or immediate surgical availability may be feasible in a tertiary referral center but difficult to implement in a smaller hospital. Therefore, guideline differences should be interpreted not only in relation to the published evidence but also in relation to referral pathways, institutional expertise, and the capacity for safe observation or timely escalation of care.

Firm guideline-based recommendations are available for several time-critical situations. Esophageal button batteries, sharp or pointed objects in the esophagus, complete esophageal obstruction, and symptomatic high-risk objects require emergent or urgent intervention. By contrast, management becomes less uniform after a high-risk object has passed beyond the esophagus, particularly in asymptomatic children. Gastric button batteries, multiple magnets beyond endoscopic reach, radiolucent objects, and water beads, therefore, remain major areas of uncertainty.

One of the most debated issues is the management of gastric button batteries in asymptomatic children. Although many batteries that have passed beyond the esophagus progress spontaneously, NASPGHAN, the 2021 ESPGHAN position paper, and Poison Control guidance use different combinations of age, battery diameter, symptoms, time since ingestion, delayed diagnosis, and radiographic progression to determine the need for removal. These recommendations should therefore be presented separately rather than merged into a single universal threshold. In practice, symptomatic children and those with selected high-risk features require urgent specialist assessment, whereas observation may be reasonable in carefully selected clinically stable patients with reliable follow-up [1,4,13,23].

Management of multiple magnets beyond endoscopic reach is another area in which recommendations remain conditional. There is broad agreement that symptomatic children, magnets associated with obstruction or perforation, and endoscopically accessible multiple magnets require early intervention. However, the optimal approach to asymptomatic distal magnets is less clearly defined. Serial clinical and radiographic surveillance may be considered in selected patients, but complications may develop despite an initially reassuring presentation. Decisions should therefore incorporate magnet number and configuration, radiographic progression, symptom evolution, access to repeated imaging, and immediate availability of pediatric surgery [1,5,7].

### 7.1. Superabsorbent Polymers: Monitoring or Early Intervention?

Superabsorbent polymer ingestion represents an emerging controversy because evidence remains limited and is derived predominantly from case reports, small case series, and retrospective observations. Unlike button batteries and magnets, no universally accepted guideline defines which asymptomatic children require endoscopic removal, hospital admission, imaging surveillance, or observation at home. The central clinical dilemma is whether to observe a child who may remain asymptomatic or to intervene before a radiolucent object progresses distally and expands beyond endoscopic reach [11,13,16,17,28,29,30].

Arguments supporting observation are based on the fact that not all ingestions lead to obstruction or require intervention. Immediate invasive management may expose an asymptomatic child to anesthesia and procedural risk, particularly when the number, product type, expansion capacity, and anatomical location are uncertain. Product heterogeneity also limits extrapolation from individual reported cases to all commercially available water beads [11,13,16,17,28,29,30].

Arguments supporting early intervention arise from the capacity of water beads to expand after ingestion, their poor visibility on conventional radiography, and the possibility of delayed intestinal obstruction. Recently witnessed ingestion, suspected gastric or proximal location, young age, multiple beads, symptoms, or exposure to a highly expandable product may favor early gastroenterological assessment. In these selected circumstances, endoscopic removal may be considered before distal progression. However, available evidence does not support routine endoscopic extraction in every asymptomatic ingestion [11,13,16,17,28,29,30].

Accordingly, the choice between observation and early intervention should be individualized rather than presented as a rigid rule. Clinically stable asymptomatic children may be considered for structured observation when follow-up is reliable, whereas vomiting, abdominal pain, distension, reduced intestinal transit, or signs of obstruction require urgent reassessment and early pediatric surgical involvement. This topic remains an area of insufficient evidence requiring prospective multicenter data and standardized reporting of product characteristics, timing, imaging findings, treatment, and outcomes.

### 7.2. Radiolucent Foreign Bodies and the Limits of Negative Imaging

Another unresolved issue is the diagnostic pathway for suspected radiolucent foreign bodies when conventional radiographs are normal. Ultrasound may identify selected objects or indirect signs of obstruction without exposing the child to ionizing radiation, but its performance is operator-dependent and varies according to object composition, size, and anatomical location [11,12,13,16,17,28,29,30]. Computed tomography may improve detection and identify perforation, inflammation, fistula formation, or obstruction, but radiation exposure limits routine use in children [6,12,31].

Even cross-sectional imaging cannot completely exclude every radiolucent object. Therefore, persistent symptoms or a strongly suggestive history should not be dismissed solely because imaging is negative. The residual clinical probability of a high-risk object, rather than the radiographic result alone, should guide decisions regarding additional imaging, diagnostic endoscopy, observation, or surgical consultation [1,2,6,12].

No single imaging algorithm is appropriate for all radiolucent foreign bodies. Plastic objects, wooden fragments, food boluses, thin bones, toothpicks, and water beads have different imaging characteristics and complication profiles. The most appropriate strategy remains individualized, and comparative pediatric studies are needed to determine the diagnostic performance and clinical utility of ultrasonography, computed tomography, magnetic resonance imaging, and endoscopic assessment in specific object categories.

### 7.3. Is There a Role for Prognostic Scores?

Although international guidelines recommend risk stratification, no universally externally validated prognostic instrument has been incorporated into routine clinical guidance for pediatric foreign body ingestion. Existing nomograms and scoring systems combine variables such as age, symptoms, pre-existing disease, object type, size, anatomical location, time since ingestion, and imaging findings, but their generalizability remains uncertain [1,2,22,24,25].

The methodological evaluation of these tools should extend beyond a single discrimination statistic. An area under the receiver operating characteristic curve indicates how well a model separates patients with and without the predicted outcome, but it does not demonstrate accurate absolute-risk estimation, clinical benefit, or transportability to another population. Calibration, decision-curve analysis, prospective impact assessment, and external validation are therefore necessary before a prognostic model can be recommended for clinical use.

Moreover, currently available tools do not all predict the same endpoint. Models estimating the need for intervention may reflect institutional thresholds, endoscopy availability, or local practice patterns, whereas models predicting complications address a different clinical question. Direct comparison is difficult when definitions of complications, intervention, treatment success, and follow-up are heterogeneous.

The relative importance of individual variables differs among study populations, and many models were developed in single centers or retrospective cohorts. Differences in referral patterns; definitions of complications; treatment thresholds; and local availability of pediatric endoscopy, anesthesia, radiology, and surgery limit direct comparison and external application [18,19,20,21,22,24,25].

Predictive tools may nevertheless be particularly valuable in intermediate-risk scenarios, in which the need for admission, urgent endoscopy, repeated imaging, or surgical consultation is not immediately clear. Future models should combine object-related, clinical, temporal, imaging, and patient-specific variables and should undergo calibration, prospective multicenter validation, comparison with clinician judgment, and assessment of clinical impact before implementation. Until then, these instruments should be regarded as decision-support tools rather than replacements for guideline-based assessment and multidisciplinary clinical judgment.

### 7.4. Prevention, Regulation, and Education

Prevention is an essential component of contemporary management because the burden of foreign body ingestion extends beyond the acute event and may include repeated procedures, prolonged follow-up, psychological distress, permanent morbidity, and substantial healthcare resource use. Button batteries, high-powered magnets, and water beads are widely available in household, electronic, recreational, and educational products, making prevention dependent on coordinated action by families, healthcare professionals, manufacturers, and regulatory authorities [4,5,11,13]. Caregiver education should emphasize safe storage, supervision, recognition of high-risk products, and immediate medical assessment after suspected ingestion. Child-resistant battery compartments, secure packaging, clear hazard labeling, and restrictions on access to high-powered magnets may reduce preventable exposure. Public information about water beads should emphasize their radiolucency, expansion capacity, potential for delayed symptom onset, and risk of intestinal obstruction [11,13,16,17]. Future studies should assess not only diagnostic and therapeutic outcomes but also the effectiveness of product redesign, packaging requirements, educational campaigns, regulatory measures, and recurrence-prevention strategies in vulnerable children. Standardized national and international reporting systems would improve epidemiological surveillance, allow comparison of complications and treatments, and support evidence-based risk communication.

## 8. Critical Synthesis

The contemporary management of gastrointestinal foreign body ingestion in children has evolved from an object-centered approach toward integrated, time-critical risk stratification. The available literature demonstrates broad agreement that object type alone is insufficient to determine management. Anatomical location, number and dimensions of the objects, symptoms, elapsed time, imaging findings, endoscopic accessibility, and patient-specific vulnerability must be assessed together to distinguish emergent intervention, urgent removal, and structured observation [1,2,3,4,5,6,7,8].

The strongest consensus applies to clearly time-critical situations. Esophageal button batteries, sharp or pointed objects in the esophagus, complete esophageal obstruction, and symptomatic multiple-magnet ingestion require rapid intervention because therapeutic delay may substantially increase the risk of necrosis, perforation, fistula formation, obstruction, and injury to adjacent structures. In these scenarios, recommendations from major pediatric society documents are relatively consistent, despite the predominantly observational nature of the supporting evidence [1,2,4,5]. Even in these high-risk scenarios, the urgency of intervention is supported predominantly by convergent society recommendations, biological plausibility, observational data, and the severity of potential harm rather than by randomized comparative trials.

Clinical decision-making is less uniform in intermediate-risk scenarios. Gastric button batteries in asymptomatic children, multiple magnets beyond endoscopic reach, distal sharp objects, radiolucent foreign bodies, and water bead ingestion remain influenced by differences among guidance documents; limited prospective evidence; and variable access to pediatric imaging, endoscopy, anesthesia, and surgery. Consequently, rigid universal thresholds are not appropriate in every case, and conditional recommendations must be interpreted within the individual clinical and institutional context [1,2,4,5,11,12,13,16,17]. This variability reflects not only gaps in evidence but also differences in publication date, document scope, procedural thresholds, and the resources assumed to be available within the healthcare system. Recommendations in these settings should therefore be presented as conditional and context-dependent rather than as universally applicable thresholds.

Time is a central component of risk, but it should not be interpreted as a single universal threshold. The clinical significance of delay differs according to the mechanism of injury: esophageal button batteries may cause rapid electrochemical tissue damage, multiple magnets may produce progressive pressure necrosis across bowel loops, and superabsorbent polymers may lead to delayed obstruction after distal progression and expansion. Therefore, time must always be integrated with object type, anatomical location, symptoms, and evidence of progression or complications [4,5,11,13,15].

The literature also supports a shift from isolated risk factors toward multidimensional assessment. Object characteristics, patient age, pre-existing disease, clinical manifestations, radiographic progression, and delayed presentation have been incorporated into nomograms and pediatric scoring systems. However, these tools currently show only moderate discriminatory performance, have been evaluated in limited populations, and lack broad external validation. They should therefore be regarded as potential decision-support instruments rather than replacements for guideline-based clinical judgment [18,21,22,24,25].

An additional contribution of the current evidence is the recognition that vulnerable pediatric populations require a modified diagnostic and preventive approach. Children with neurodevelopmental disorders, communication difficulties, pica, recurrent ingestion, gastrointestinal abnormalities, or intentional ingestion may present late, provide an incomplete history, or experience repeated episodes. In these groups, a lower threshold for investigation, multidisciplinary assessment, mental health or developmental support, and structured recurrence prevention may be appropriate [26,27].

Taken together, the evidence supports a practical framework in which management is organized around three sequential questions: whether the object poses an immediate mechanism of injury, whether it is endoscopically accessible or requires surgical involvement, and whether reliable observation is clinically and logistically feasible. This framework does not eliminate uncertainty but offers a structured way to integrate guideline recommendations with object-related, temporal, clinical, imaging, and patient-specific variables. Its practical performance and generalizability require prospective evaluation.

Future progress will depend on prospective multicenter registries; standardized definitions of complications and therapeutic success; and harmonized reporting of object characteristics, symptoms, imaging findings, elapsed time, interventions, and outcomes. Predictive models should be developed using adequately sized and representative pediatric cohorts, undergo robust internal validation, and subsequently be evaluated in independent external populations and different healthcare systems. Direct head-to-head comparisons should assess discrimination, calibration, clinical utility, feasibility, and performance relative to guideline-based clinical judgment. Particular research priorities include radiolucent foreign bodies, water beads, distal magnets, post-removal surveillance after esophageal button battery injury, and recurrence prevention in vulnerable pediatric populations.

## 9. Strengths and Limitations

This review has several strengths. It integrates, within a single clinically oriented framework, the main high-risk categories of pediatric gastrointestinal foreign body ingestion, including button batteries, multiple magnets, sharp objects, radiolucent materials, and superabsorbent polymers. It also compares major international society recommendations; emphasizes time-critical decision-making; incorporates patient-related vulnerability and predictive variables; and proposes a clinically oriented conceptual framework that differentiates emergent intervention, urgent management, selected early intervention, and structured observation. The inclusion of recent literature and the specific analysis of vulnerable pediatric populations further increase the clinical relevance of the review.

Several limitations should also be acknowledged. First, despite the use of a structured search strategy and explicit eligibility criteria, this study remains a narrative review rather than a formal systematic review. Study selection and prioritization were guided by clinical relevance and contribution to the predefined topics, which may have introduced selection bias and may have resulted in the omission of relevant evidence. In addition, screening and evidence interpretation were not performed through a formal independent dual-review process. No standardized risk-of-bias instrument, certainty-of-evidence framework, or quantitative evidence synthesis was applied because the included literature was methodologically heterogeneous and comprised guidelines, position papers, reviews, observational studies, and selected clinically informative reports. Consequently, the relative methodological quality of individual studies was not formally graded, and the conclusions should be interpreted as a clinically oriented synthesis rather than as estimates derived from a systematic evidence assessment. To reduce overinterpretation, recommendations are distinguished throughout the manuscript according to whether they reflect broad guideline agreement, conditional recommendations, observational evidence, or expert consensus.

Second, the available evidence is dominated by retrospective studies, single-center cohorts, case series, and expert consensus. Randomized or prospective comparative studies are scarce, particularly for rare but high-risk scenarios. The quality and strength of recommendations therefore vary substantially among object categories and clinical situations. Accordingly, the narrative descriptors used in this review indicate the degree of consistency and clinical support but do not represent formal certainty-of-evidence ratings.

Third, definitions of complications, intervention thresholds, treatment success, and follow-up differ across studies. Variation in healthcare systems and in the availability of pediatric endoscopy, anesthesia, radiology, surgery, and intensive care may also limit the generalizability of published recommendations and predictive models. Accordingly, apparent differences between guideline recommendations may partly reflect healthcare-system context and should not always be interpreted as purely evidence-based disagreement.

Fourth, the evidence regarding radiolucent foreign bodies and water beads remains particularly limited. Most publications on superabsorbent polymers consist of case reports or small case series, and no universally accepted management protocol or validated prognostic criteria are currently available [11,13,16,17,28,29,30,31].

Finally, existing predictive models and scoring systems have generally been developed in retrospective or single-center populations and lack broad external validation. Their discriminatory performance, calibration, and clinical impact across different pediatric settings remain uncertain. Accordingly, these tools should be considered exploratory decision-support instruments rather than definitive standards of care [22,24,25].

The proposed management flowchart represents a conceptual synthesis of existing recommendations and reviewed evidence. It has not been prospectively validated, tested for interobserver agreement, or evaluated for its impact on clinical outcomes. Therefore, it should be interpreted as an educational and decision-support framework rather than as a validated clinical pathway or formal guideline.

Despite these limitations, the review provides an updated and integrated synthesis of current evidence and identifies clinically relevant priorities for prospective multicenter research, standardized outcome definitions, transparent predictive-model development, independent external validation, direct model comparison, and prospective evaluation of clinical impact.

## 10. Declaration Regarding the Use of Artificial Intelligence

During the preparation of this manuscript, the authors used ChatGPT (version 5.5, OpenAI; accessed in July 2026) to assist with restructuring and organizing sections of the manuscript, improving linguistic clarity and coherence, rephrasing selected passages, and supporting the organization and standardized presentation of tables and conceptual figures. The artificial intelligence tool was not used to generate clinical or research data; perform statistical analyses; independently interpret study findings; formulate scientific conclusions or clinical recommendations; or create or modify clinical, radiological, endoscopic, histological, or other primary research images. The selection and verification of bibliographic sources, interpretation of the literature, validation of the scientific content, and final design of the tables and figures were performed by the authors. The authors critically reviewed and verified the entire manuscript and assume full responsibility for its accuracy, integrity, and final form.

## 11. Conclusions

Pediatric gastrointestinal foreign body ingestion encompasses a broad clinical spectrum, ranging from spontaneous passage of small inert objects to rapidly progressive, life-threatening injury caused by button batteries, multiple high-powered magnets, sharp or pointed objects, and superabsorbent polymers. Contemporary management should therefore move beyond identification of the ingested object and incorporate a time-critical, risk-stratified assessment based on object type, number and dimensions, anatomical location, symptoms, imaging findings, elapsed time, endoscopic accessibility, and patient-specific vulnerability. The strongest international consensus applies to clearly time-critical situations, including esophageal button batteries, sharp objects in the esophagus, complete esophageal obstruction, and symptomatic high-risk ingestions. These cases require emergent or urgent intervention. By contrast, gastric button batteries, distal magnets, radiolucent objects, distal sharp objects, and water beads often require individualized decision-making because recommendations are less uniform and the supporting evidence is predominantly observational.

A normal radiograph does not exclude ingestion when the suspected object is radiolucent or when clinical suspicion remains high. In such cases, further evaluation with ultrasonography, computed tomography, diagnostic endoscopy, or surgical consultation should be selected according to symptoms and the estimated risk of complications. Multidisciplinary management is particularly important when perforation, fistula formation, obstruction, vascular injury, or severe post-extraction tissue damage is suspected. Predictive models and clinical scoring systems may support future risk stratification, but current tools remain insufficiently externally validated and should not replace guideline-based clinical judgment. Children with neurodevelopmental disorders, pica, recurrent ingestion, communication difficulties, gastrointestinal abnormalities, or intentional ingestion require a lower threshold for investigation, multidisciplinary assessment, and structured recurrence prevention.

The strongest recommendations apply to clearly time-critical scenarios supported by broad agreement among major pediatric guidance documents. In intermediate-risk situations, management should remain individualized because the available evidence is predominantly observational and recommendations are frequently conditional or context-dependent.

Prospective multicenter studies, standardized definitions of complications and therapeutic outcomes, transparent development and independent external validation of predictive models, direct comparisons between available tools, and prospective assessment of their clinical impact are needed before prognostic instruments can be integrated into routine pediatric care. Stronger preventive and regulatory strategies are also required to reduce avoidable morbidity.

## Figures and Tables

**Table 1:** Comparison of NASPGHAN and ESGE/ESPGHAN recommendations for the initial diagnostic evaluation of pediatric foreign body ingestion [1,2].

Assessment Element	NASPGHAN 2015	ESGE/ESPGHAN 2017	Practical Interpretation
Initial radiographic evaluation	Radiographic evaluation is recommended when ingestion of a radiopaque foreign body is suspected. The radiographic field should be selected according to the suspected location and type of object.	Early radiographic evaluation is recommended in children with suspected foreign body ingestion.	Radiography remains the first-line investigation for suspected radiopaque objects. The examined anatomical region should include the expected location of the foreign body.
Radiographic views	Additional views may be required to determine object number, location, orientation, and characteristics, particularly when a button battery, multiple magnets, or a sharp object is suspected.	Anteroposterior and lateral radiographs are recommended when necessary for localization and characterization.	Two-view radiography is especially useful when differentiation between a coin and a button battery is required or when the apparent number of magnets is uncertain.
Coin–button battery differentiation	The double-ring or halo appearance on the anteroposterior view and the step-off appearance on the lateral view support the diagnosis of a button battery.	Similar radiographic features are used to distinguish button batteries from coins.	A suspected button battery should be treated as a time-critical emergency until its location is confirmed.
Radiolucent foreign bodies	A negative radiograph does not exclude ingestion. Further evaluation should depend on symptoms, history, and clinical suspicion; endoscopy or other imaging may be required.	CT may be considered for suspected radiolucent foreign bodies, particularly when the diagnosis remains uncertain or complications are suspected.	Additional investigation should be individualized. Negative radiographs should not delay further evaluation in a symptomatic child or when suspicion remains high.
Role of computed tomography	CT may be useful when perforation, fistula, obstruction, or injury to adjacent structures is suspected, but radiation exposure should be considered.	CT may be considered for radiolucent objects and suspected complications.	CT is not routinely indicated and should be reserved for selected cases in which its findings are likely to alter management.
Assessment for complications	Clinical and imaging findings should be integrated when obstruction, perforation, fistula, or vascular injury is suspected.	Further cross-sectional imaging is recommended or considered when complications are suspected.	Signs of perforation, obstruction, mediastinal involvement, or vascular injury require immediate multidisciplinary assessment.

Abbreviations: CT, computed tomography; ESGE, European Society of Gastrointestinal Endoscopy; ESPGHAN, European Society for Paediatric Gastroenterology, Hepatology and Nutrition; NASPGHAN, North American Society for Pediatric Gastroenterology, Hepatology and Nutrition.

**Table 2:** Practical role of imaging modalities according to the suspected foreign body type [4,7,8,11].

Foreign Body Type	Radiography	Ultrasound	CT	Comments
Coin	+++	−	−	Usually readily identified on radiographs; additional imaging is rarely required.
Button battery	+++	−	++	The double-ring or halo sign and the lateral step-off appearance may differentiate a button battery from a coin. CT is reserved for suspected complications.
Magnets	+++	+/−	++	Anteroposterior and lateral radiographs help determine the number, location, and progression of the magnets. CT may be required when complications are suspected.
Water beads	−	++	++	Usually radiolucent. Ultrasonography may identify the beads or signs of obstruction; CT may be considered when the diagnosis is uncertain or complications are suspected.
Plastic or wooden objects	− or +/−	+/−	++	Visibility depends on material, size, thickness, and anatomical location. Further imaging should be guided by symptoms and clinical suspicion.
Food bolus or thin fish/chicken bones	+/−	+/−	++	Imaging sensitivity is variable. Persistent symptoms despite negative radiographs may require endoscopic evaluation or additional imaging.

Interpretation of symbols: +++, usually readily detectable or routinely useful; ++, useful in selected cases; +/−, variable or limited usefulness; −, generally not detectable or not routinely useful. Abbreviations: CT, computed tomography.

**Table 3:** Time-critical management recommendations for high-risk pediatric foreign body ingestion according to major international society documents [1,2,4,5,8].

Clinical Situation	NASPGHAN 2015	ESGE/ESPGHAN 2017 and Specific ESPGHAN Position Papers	Evidence/Source Interpretation
**Button battery in the esophagus**	Emergent endoscopic removal, ideally within 2 h.	Immediate endoscopic removal is recommended; the 2021 ESPGHAN position paper emphasizes the risk of rapid tissue injury.	Broad consensus; evidence is mainly observational.
**Button battery in the stomach, asymptomatic patient**	Clinical and radiographic surveillance may be appropriate. Removal should be considered according to symptoms, age, battery size, delayed diagnosis, associated magnet ingestion, and lack of progression.	Individualized management is recommended; removal is favored in symptomatic patients and selected high-risk situations.	Conditional recommendations: differences exist between guidance documents.
**Multiple magnets in the gastrointestinal tract**	Urgent endoscopic removal if accessible. If beyond endoscopic reach, serial clinical and radiographic monitoring and early surgical assessment are recommended.	Similar approach; the 2023 ESPGHAN position paper emphasizes early intervention and close surgical involvement.	Expert consensus and retrospective observational evidence.
**Sharp object in the esophagus**	Emergent endoscopic removal because of the risk of perforation and injury to adjacent structures.	Emergent endoscopic removal is recommended, including in asymptomatic children.	Strong clinical consensus; limited comparative evidence.
**Sharp object in the stomach or duodenum**	Endoscopic removal if accessible and safe; otherwise, close clinical and radiographic monitoring.	Urgent endoscopic removal, generally within 24 h.	Recommendations are based mainly on observational evidence and expert consensus.
**Coin in the esophagus**	Emergent removal in symptomatic children unable to manage secretions or with respiratory symptoms; otherwise, removal within 24 h.	Emergent removal in symptomatic children and urgent removal within 24 h in asymptomatic children.	Broad agreement between guidelines.

Abbreviations: ESGE, European Society of Gastrointestinal Endoscopy; ESPGHAN, European Society for Paediatric Gastroenterology, Hepatology and Nutrition; NASPGHAN, North American Society for Pediatric Gastroenterology, Hepatology and Nutrition.

**Table 4:** Management of button and cylindrical battery ingestion in children [1,4,14,15].

Clinical Scenario	Recommended Management
Button battery impacted in the esophagus	Emergent endoscopic extraction immediately after diagnosis, preferably in <2 h.
Unstable patient or significant gastrointestinal bleeding	Emergent management in an experienced center with multidisciplinary approach and surgical and vascular availability.
Suspected vascular injury or severe esophageal injury adjacent to major vessels	Vascular imaging by CT angiography or MR angiography and multidisciplinary management.
Suspected or confirmed esophageal button battery ingestion within the previous 12 h	Honey may be administered at a dose of 10 mL every 10 min, for a maximum of six doses, in children aged ≥12 months who can swallow safely and whose ingestion occurred within the previous 12 h. Sucralfate may be administered in the medical setting, for a maximum of three doses. Neither measure should delay emergent endoscopic removal.
Button battery in the stomach, symptomatic patient	Endoscopic evaluation and early extraction.
Gastric button battery ≥ 20 mm in child < 5 years	Consider early endoscopic removal, particularly when ingestion was recent, the child is young, the battery is ≥20 mm, or radiographic progression is absent. Timing should be individualized according to the applicable guidance document and clinical context.
Gastric button battery in an asymptomatic patient without risk factors	Clinical and radiological monitoring; management individualized according to age, size, time from ingestion and progression.
Persistent gastric battery without progression	Repeat radiographic assessment and specialist review. Endoscopic removal should be considered when the battery does not progress, particularly in young children, for batteries ≥ 20 mm, after delayed diagnosis, or if symptoms develop.
Battery distal to the stomach, asymptomatic patient	Clinical monitoring and confirmation of passage; radiological reassessment if passage is not confirmed or symptoms occur.
Battery distal to the stomach, symptomatic patient	Urgent evaluation for complications and gastroenterology/surgical consultation.
Cylindrical battery in the esophagus	Emergent endoscopic extraction.
Cylindrical battery in the stomach, asymptomatic patient	Clinical and radiographic monitoring may be appropriate. Endoscopic removal should be considered if the battery casing is damaged, symptoms develop, gastric retention is prolonged, or radiographic progression is absent.
Cylindrical battery distal to the stomach	Clinical and radiological monitoring; intervention if there is lack of progression or symptoms/complications occur.

Note: Management should be individualized according to the child’s age, battery type and diameter, anatomical location, time elapsed since ingestion, symptoms, radiographic progression, and the possibility of unwitnessed or delayed ingestion. Recommendations for gastric button batteries differ among NASPGHAN, the 2021 ESPGHAN position paper, and Poison Control guidance; therefore, no single threshold should be applied universally. Abbreviations: CT, computed tomography; MR, magnetic resonance.

**Table 5:** Management of magnet ingestion in children [1,5,7].

Clinical Scenario	Recommended Management
Single magnet, asymptomatic patient	Clinical and radiographic monitoring may be appropriate, with assessment of progression and confirmation of passage. Apparent single-magnet ingestion should be interpreted cautiously when the history is uncertain because multiple adherent magnets may mimic a single object.
Single magnet in the esophagus or stomach, endoscopically accessible	Endoscopic removal may be considered, particularly when the magnet is located in the esophagus, when the number of magnets is uncertain, when progression is absent, or when patient- or object-related risk factors are present.
Single magnet distal to the stomach, asymptomatic patient	Clinical and radiological monitoring until progression/passage; urgent reassessment if symptoms occur.
Multiple magnets or magnet plus metallic object in esophagus/stomach	Urgent endoscopic extraction if accessible because of the risk of compression between digestive segments, ischemia, necrosis, fistula or perforation.
Multiple magnets distal to the stomach, symptomatic patient	Urgent surgical consultation and intervention according to clinical and imaging status.
Multiple magnets distal to the stomach, asymptomatic patient	Hospital admission or very close outpatient surveillance with serial abdominal radiographs may be considered only in carefully selected patients, with early pediatric surgical involvement and immediate access to reassessment if symptoms develop.
Lack of progression on serial radiographs	Multidisciplinary reassessment; endoscopic or surgical intervention considered according to location, accessibility and clinical context.
Signs of complications: persistent abdominal pain, vomiting, fever, acute abdomen, pneumoperitoneum, obstruction or suspected perforation/fistula	Immediate pediatric surgical assessment and operative management according to the clinical and imaging findings.
Endoscopy impossible or unsuccessful in multiple magnets/associated metallic object	Transfer to a specialized center or urgent surgical consultation.

Note: The absence of symptoms at presentation does not exclude severe gastrointestinal injury, particularly after ingestion of multiple magnets or a magnet together with another metallic object. Management should be individualized according to the number, location, configuration, radiographic progression, elapsed time since ingestion, symptoms, and endoscopic accessibility. Recommendations are based predominantly on society guidance, expert consensus, and retrospective observational evidence. Abbreviation: ESPGHAN, European Society for Paediatric Gastroenterology, Hepatology and Nutrition.

**Table 6:** Pragmatic management of water bead ingestion in children [11,13,16,17].

Clinical Scenario	Recommended Management
Suspected or confirmed ingestion, asymptomatic patient	Detailed history regarding timing, estimated number, product type, and possible expansion capacity; clinical assessment and caregiver education regarding alarm symptoms. Management should be individualized according to age, ingestion context, and reliability of follow-up.
Recent ingestion, water beads suspected or identified in the stomach	Early gastroenterology evaluation; endoscopic removal may be considered in selected cases before distal progression and substantial expansion, particularly in young children, symptomatic patients, suspected multiple ingestion, or exposure to highly expandable products.
Normal abdominal radiograph but persistent clinical suspicion	A normal radiograph does not exclude ingestion. Consider ultrasonography and, in selected cases, cross-sectional imaging according to symptoms, diagnostic uncertainty, and suspected complications.
Water beads distal to the stomach, asymptomatic patient	Close clinical observation may be considered in carefully selected, clinically stable patients. The need for admission and additional imaging should be individualized, with clear caregiver instructions and a low threshold for reassessment if symptoms develop.
Persistent vomiting, abdominal pain or distension, reduced intestinal transit or other signs of obstruction	Urgent evaluation for intestinal obstruction with appropriate imaging and early surgical consultation.
Clinical or imaging signs of intestinal obstruction	Urgent pediatric surgical assessment; operative management should be individualized according to the location, severity, and clinical stability of the patient.
Suspected perforation, intestinal ischemia, peritonitis or clinical deterioration	Emergency surgical assessment; imaging must not delay intervention in unstable patients or acute abdomen.
Uncertain diagnosis in a young child with obstructive symptoms and unwitnessed ingestion	Water beads should be included in the differential diagnosis; history should be redirected toward access to toys or products containing superabsorbent polymers.

Note: Recommendations for water bead ingestion are based predominantly on case reports, small case series, and expert opinion. No validated prognostic criteria or universally accepted management protocol are currently available.

**Table 7:** Clinical characteristics and associated risks in pediatric populations at increased risk of foreign body ingestion [26,27].

Patient Category	Clinical Characteristics	Associated Risks
Autism spectrum disorders	Communication difficulties, repetitive behaviors, persistent oral exploration	Unwitnessed ingestion, delayed diagnosis, repeated episodes.
Global developmental delay/intellectual disability	Difficult history, reduced cooperation	Unwitnessed ingestions, multiple objects, delayed diagnosis.
Pica disorder	Repeated ingestion of non-food objects	Increased risk of recurrence and multiple ingestions.
Adolescents with psychiatric disorders/voluntary ingestion	Voluntary ingestion, self-harming behavior	Multiple ingestions, need for psychiatric evaluation.
History of repeated foreign body ingestion	History of repeated episodes	Need for recurrence prevention and structured long-term follow-up.

## Data Availability

No new data were created or analyzed in this study. Data sharing is not applicable to this article.

## Abbreviations

The following abbreviations are used in this manuscript:

ASGE	American Society for Gastrointestinal Endoscopy
CT	Computed tomography
ESGE	European Society of Gastrointestinal Endoscopy
ESPGHAN	European Society for Paediatric Gastroenterology, Hepatology and Nutrition
MRI	Magnetic resonance imaging
NASPGHAN	North American Society for Pediatric Gastroenterology, Hepatology and Nutrition
SAP	Superabsorbent polymers
